# Women’s Lives Matter—The Critical Need for Women to Prioritize Optimal Physical Activity to Reduce COVID-19 Illness Risk and Severity

**DOI:** 10.3390/ijerph181910271

**Published:** 2021-09-29

**Authors:** Karla P. Garcia-Pelagio, Tamara Hew-Butler, Mariane M. Fahlman, Joseph A. Roche

**Affiliations:** 1 Departamento de Física, Facultad de Ciencias, Universidad Nacional Autónoma de México, Ciudad de México 4510, Mexico; 2 Division of Kinesiology, Health and Sport Studies, College of Education, Wayne State University, Detroit, MI 48201, USA; tamara.hew-butler@wayne.edu (T.H.-B.); m.fahlman@wayne.edu (M.M.F.); 3 Physical Therapy Program, Eugene Applebaum College of Pharmacy and Health Sciences, Wayne State University, Detroit, MI 48201, USA

**Keywords:** physical activity (PA), exercise, health and wellness, women’s health, preventive healthcare, vaccination, risk mitigation, nonpharmacological interventions

## Abstract

Physical activity (PA) is beneficial for the health and wellness of individuals and societies. During an infectious disease pandemic, such as the one caused by COVID-19, social distancing, quarantines, and lockdowns are used to reduce community spread of the disease. Unfortunately, such nonpharmacological interventions or physical risk mitigation measures also make it challenging to engage in PA. Reduced PA could then trigger physiological changes that affect both mental and physical health. In this regard, women are more likely to experience physical and psychological distress. PA is a safe and effective nonpharmacological modality that can help prevent and manage several mental and physical health problems when performed correctly. PA might even confer benefits that are directly related to decreasing COVID-19 morbidity and mortality in women. In this review, we summarize why optimal PA must be a priority for women during the COVID-19 pandemic. We then discuss chronic COVID-19 illness and its impact on women, which further underscores the need for worldwide preventive health strategies that include PA. Finally, we discuss the importance of vaccination against COVID-19 for women, as part of prioritizing preventive healthcare and an active lifestyle.

## 1. Introduction

The World Health Organization (WHO) declared coronavirus disease 2019 (COVID-19) a pandemic on 11 March 2020 [1,2]. To control community spread of the COVID-19 pathogen (severe acute respiratory syndrome coronavirus 2, SARS-CoV-2), most countries around the world issued mandates and guidelines for social distancing, which included staying ~two meters (~six feet) apart from other people [3] and not gathering in groups [4,5,6]. Unfortunately, a negative consequence of such necessary measures to control the spread of an infectious disease is that, it creates barriers to engaging in sufficient amounts of physical activity (PA) [7], thus predisposing societies to a “pandemic of physical inactivity” [8]. Reduced PA, coupled with reduced social interaction and changes in work and living arrangements due to the COVID-19 pandemic, has negatively impacted the health and wellness of individuals and communities [9]. The negative effects of social distancing and isolation range from mental health concerns, such as anxiety and depression, to disturbances in physical health in the form of metabolic changes, increased adiposity, and multisystem deconditioning (e.g., negative changes in the cardiopulmonary, neuromuscular, and musculoskeletal systems) [10,11]. According to the United Nations and The World Economic Forum, data from the Ebola and Zika epidemics indicate that, during epidemics, women are more vulnerable to the effects of both the disease and quarantine due to their pivotal roles both at home and on the front lines of healthcare and the economy [12].

The need for adequate PA during the COVID-19 pandemic has been discussed for various special populations (e.g., aging [13], cancer [14], arthritis [7], congenital heart disease [15], and diabetes [16]). However, our examination of the literature suggests that the importance of optimal PA for women during the COVID-19 pandemic has only been minimally highlighted [17]. This is unfortunate because women make up ~70% of frontline workers in the healthcare and social services sectors, making them particularly vulnerable to COVID-19 exposure [18]. Since PA is a safe, effective, and simple nonpharmacological approach for improving health and wellness [19,20,21], it is imperative that the potential benefits of PA for women during the COVID-19 pandemic are examined and described. The benefits of PA are derived from both local effects on the musculoskeletal and neuromuscular systems (e.g., improved cardiorespiratory fitness, muscle strength and endurance, flexibility, and neuromotor control) as well as systemic effects (e.g., improved circulation, immune system function, insulin sensitivity and other endocrine functions, and mental health) [20,21,22,23]. Thus, PA might have numerous benefits for women during the COVID-19 pandemic.

In this era of COVID-19, even with vaccines and therapeutics, some physical risk mitigation measures (distancing, masking, hygiene, and self-isolation when sick) are likely to be necessary until COVID-19 community spread becomes insignificant [24,25]. Mathematical models and emerging data suggest that premature relaxation of physical risk mitigation measures might result in new waves of infections [24,25,26,27,28]. As we work to exit the COVID-19 pandemic, it is imperative that the barriers to PA and their effect on health and wellness are further investigated [8], and messages regarding the critical need for optimal PA are amplified by public health agencies [21,29].

**Figure 1:**
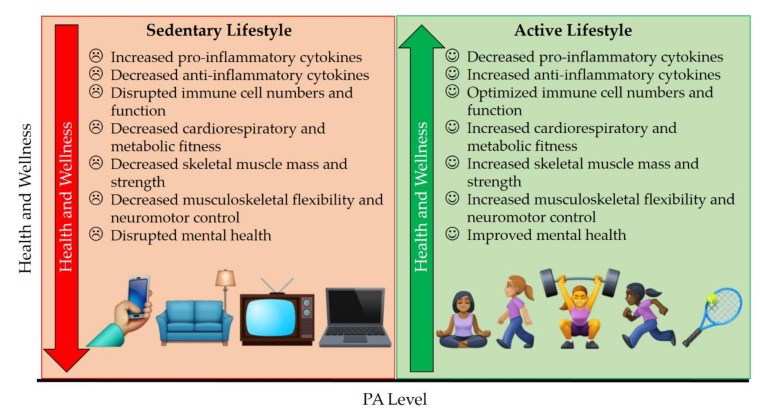
Health benefits of an active lifestyle. Being physically active rather than sedentary has multiple beneficial effects and improves overall health and wellness. Due to positive modulatory effects on multiple physiological systems, being physically active during the COVID-19 pandemic can be beneficial for healthy women, as well as for women who might have asymptomatic or uncomplicated COVID-19.

**Figure 2:**
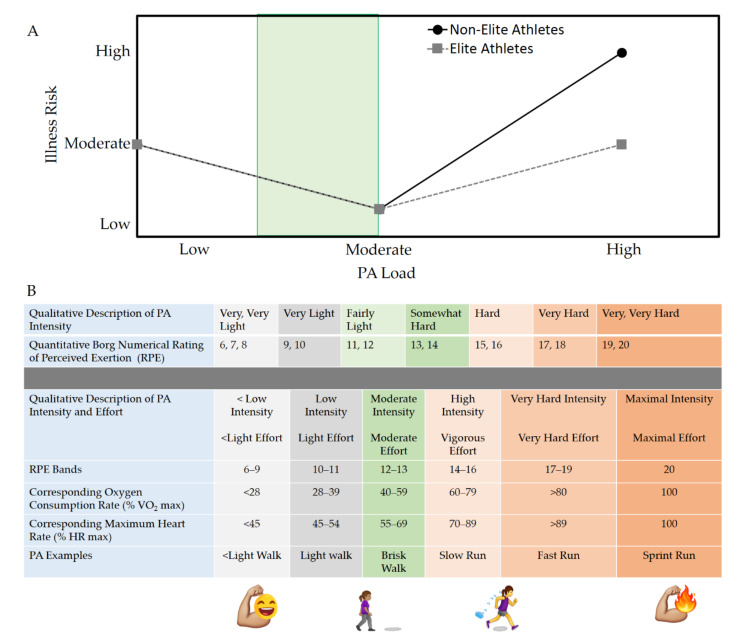
“Just Right” PA and reduction in overall illness risk. (**A**) Since there is a link between PA load (the combination of activity intensity and duration) and illness risk, it is important not to perform high loads of unaccustomed PA during the COVID-19 pandemic [57]. For the majority of women in the population whose physical ability level is not at the level of elite athletes, moderate PA is likely to be beneficial while unaccustomed, intense PA is likely to be harmful. Each individual should gauge for themselves, under the advice of suitable healthcare professionals, what their “just right” level of PA is (Green Zone in figure). For elite athletes, while setting PA load, the risk of illness must be weighed against the benefit of being able to return to a competitive level when COVID-19 restrictions are lifted. Furthermore, healthy elite athletes who are accustomed to high PA loads as part of systematic training have a lower risk of illness even with high PA loads. (**B**) Since the self-reported rating of perceived exertion (RPE) correlates well with VO_2_ max and HR, RPE is a simple yet reliable tool for gauging the intensity of PA and for adjusting PA load as needed [58,59].

**Figure 3:**
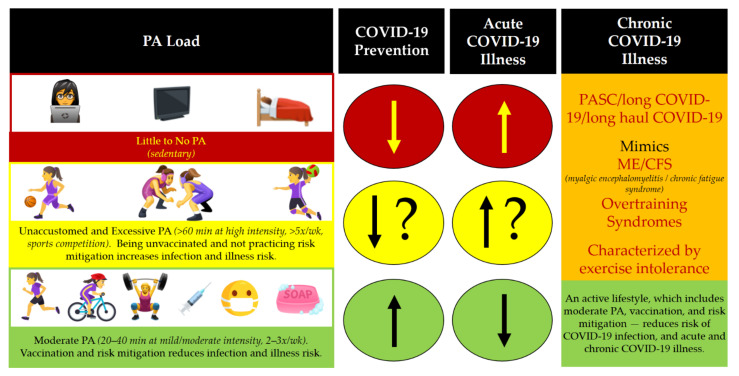
PA, COVID-19 infection and illness, and population health. Following an active lifestyle, which prioritizes preventive healthcare in the form of moderate levels of PA, vaccination when eligible, and continued COVID-19 risk mitigation, would likely help women in optimizing their mental and physical health and reduce their risk of COVID-19 infection and illness. The health of women in societies is of critical importance during the COVID-19 pandemic due to the pivotal roles women play in commerce, healthcare, and family health and wellness.

In this review, we provide an overview of the mental and physical health benefits of PA for women during the COVID-19 pandemic (Figure 1). We also provide a word of caution on the risks associated with over-exercising and emphasize the need to adjust PA load according to one’s own ability (Figure 2). We then discuss the pathogenesis of chronic COVID-19 illness (post-acute sequelae of SARS-CoV-2 infection, PASC; a.k.a. “long COVID”) [30], which may disproportionately affect women (Figure 3) [31]. Finally, we acknowledge the optimism that has been ushered in by safe and effective COVID-19 vaccines, which have received approval or emergency use authorization across the world. We then make the case that, similar to how PA load should be adjusted to reduce the risk of COVID-19 infection and complications, it would be advisable to adjust PA load before and after COVID-19 vaccination in order to reduce the risk of extremely rare adverse events associated with vaccination and to provide the body with the time it needs to restore homeostasis following vaccine-induced perturbation of the immune, muscular, and other systems.

We acknowledge that the gender and biological sex of a person are related but not synonymous [32,33,34]. In this paper, the terms “women/females” and “women’s health” refer to biological females and their specific health considerations, respectively. However, the information presented here may be useful for both biological females and males, as well as individuals of diverse genders [35]. We also clarify that in this review, where appropriate, we have used the more inclusive term physical activity (PA) rather than the specific term “exercise” because exercise is a subtype of PA, which must meet certain precise criteria [36].

## 2. Mental Health Benefits of PA

To assess the impact of COVID-19 on women, vast sex-disaggregated data will have to be collected and analyzed [18,37,38]. Based on recent COVID-19 reports and experience from past MERS and SARS outbreaks, it is known that women face specific risks due to social environments, norms, and unequal power relations, making them highly vulnerable to psychological and physical distress [12,39,40]. During long periods of social isolation, it is common for people to experience symptoms of depression, such as sadness, loss of interest or pleasure, feelings of guilt or low self-worth, disturbed appetite or sleep, tiredness, poor concentration, and suicidal thoughts [40]. In particular, women are twice as likely to develop anxiety disorders and mental health crises when compared to men, presumably because of the effect of sex hormones such as estradiol and progesterone [39]. During COVID-19-related quarantines, data suggest that there has been an increase in mild depression, stress, and anxiety reported in women [41]. These changes in mental health might manifest as stress-related eating or using psychoactive substances (e.g., drugs, alcohol, and nicotine) [40].

Longitudinal and cross-sectional studies have demonstrated the positive impact of regular PA on depression [42]. Specifically, PA undertaken before an emotionally stressful stimulus reduces the magnitude of immediate stress and the consumption of unhealthy foods [43]. What is highly encouraging is that PA reduces sleep disorders, anxiety, and depression, and psychoactive substance cravings, even after a single session [44,45,46]. Such improvements in mental health and cognition have been linked to changes in the prefrontal area of the cerebral cortex [47,48]. Acute PA (>60% maximum oxygen uptake) releases beta-endorphin (an endogenous peptide), which modulates pain, reduces stress, activates reward and pleasure areas in the brain, and stabilizes mood and behavior through its agonistic effects on opioid receptors [42,49]. Due to mandates and guidelines for social distancing during the COVID-19 pandemic, mental health professionals have been required to use telemedicine to provide consultation and prescribe cognitive, behavioral, and pharmacotherapies to treat mental health conditions [50,51,52]. Given that in-person psychotherapy might be challenging in terms of scheduling due to social distancing, the therapeutic use of PA to improve mental health is even more appropriate [53].

## 3. Physical Health Benefits of PA

The American College of Sports Medicine recommends paying attention to four domains of PA, namely cardiorespiratory fitness, muscular fitness (strength and endurance), flexibility, and neuromotor control [23]. PA that covers these four domains has been shown to produce local effects within cardiac and skeletal muscle, as well as systemic effects in all other physiological systems in the body in both women and men [10,21,23]. Specifically, the local and systemic benefits of PA are relevant to maintaining an optimal body mass index (BMI), possessing better insulin sensitivity, achieving a healthy blood lipid profile, and avoiding high blood pressure, which collectively reduces the risk of heart disease—the leading cause of death globally according to the WHO [54,55,56].

Cardiorespiratory fitness (CRF) and the maximal intensity of PA that a person is capable of performing are biomarkers of cardiovascular health. The gold standard measure of CRF is maximal oxygen consumption (VO_2_ max), which is the product of cardiac output and the arteriovenous oxygen concentration difference during increasingly demanding PA [60,61,62,63]. In more informal clinical or at-home settings, CRF and PA intensity may be assessed as a rating of perceived exertion (RPE) on a scale of 6–20 or 0–10 [58,59,64,65]. Since VO_2_ max, heart rate (HR), and RPE (on a 6–20 scale) are well correlated, it is possible for a person to assess their PA intensity based on RPE and to use RPE as a guide to engage in optimal PA based on their own capacity [58] (Figure 2B). The promising aspect of using RPE to adjust PA intensity is not only its simplicity but also its ability to account for HR changes, which may be caused by cardiovascular medications [66,67,68].

When compared to age-matched men, women have lower VO_2_ max levels due to physiological factors, such as reduced ventricular ejection fraction, hemoglobin concentration, muscle mass, and higher body fat percentages [21,23,69,70]. Even though women have lower CRF compared to men prior to menopause, they have a lower risk of mortality from cardiovascular disease, possibly because hormones such as estradiol and progesterone play protective roles [49,55,60,71]. However, since those at highest risk for COVID-19 complications are over the age of 65 years, women >65 years who contract COVID-19 are most likely to be postmenopausal and not have the benefit of premenopausal cardioprotection [72]. Other factors that negatively impact COVID-19 outcomes are hypertension, cardiovascular disease, diabetes, and obesity [73,74]. Since these preexisting conditions are positively impacted by CRF, optimizing cardiorespiratory function through PA might most likely be beneficial in the context of preventing COVID-19 complications in women [8,71]. Furthermore, since better CRF correlates with optimal functioning of the immune system and its inflammatory responses, it is likely that improved CRF might even have direct benefits in the context of COVID-19 [75] (Figure 3).

While excessive PA can be detrimental to health in untrained individuals, several studies have shown that moderate PA has a modulatory effect on the immune system and inflammation. Depending on regularity, type, duration, and intensity, PA can have pro- or anti-inflammatory downstream effects [76,77,78]. The balance between these opposing effects is important because immune responsiveness determines whether PA is beneficial or detrimental (e.g., improperly dosed PA can result in muscle injuries or, even worse, rhabdomyolysis and renal failure) [79]. Therefore, there is a dose–response relationship between PA and health outcomes (Figure 2). PA can induce changes in peripheral blood cell numbers, granulocyte activity, natural killer (NK) cells, lymphocytes, and plasma cytokine profiles, which correlate with improvements in outcomes of physical health [80]. Angiotensin converting enzyme 2 (ACE2), which is a plasma membrane protein, acts as an entry point for SARS-CoV-2 into host cells and also undergoes changes with PA that might confer a protective effect on the organ systems affected by COVID-19 [81,82]. However, unaccustomed, intense, and prolonged PA can cause tissue damage, impair the ability of the immune system to respond appropriately to an immune challenge (due to lymphopenia), trigger excessive inflammation, and even result in immunosuppression [80,83]. The effects of excessive PA (e.g., prolonged and repetitive high-intensity activity) (Figure 2) can result in physiological changes that resemble sepsis, albeit with milder symptoms [80,84]. The benefits of optimal PA, however, might not just improve overall health and wellness in women, but might also have direct benefits related to decreasing COVID-19 morbidity and mortality that go beyond the natural biological advantages of the female sex in the context of COVID-19 [71,74,85,86,87,88]. Thus, it could be argued that, optimal PA might be one of the most effective strategies for women and for society in general to stay healthy during the COVID-19 pandemic [54,89]—indeed, there is already evidence supporting this notion. One retrospective observational study of over 48,000 patients found that those who were more physically active in the two years preceding COVID-19 infection had reduced odds for hospitalization and death due to COVID-19 [90]. Another study of over 76,000 adults found that those who engaged in regular strength training and aerobic PA were less likely to become infected with COVID-19, and those that were infected were less likely to die [91].

The emerging evidence suggests that patients who develop COVID-19 complications have an abnormal immune response [92], which includes lymphocytopenia (in ~83%), thrombocytopenia (in ~36%), leukopenia (in ~33%), and elevated levels of c-reactive protein (CRP, in ~58%) [93,94]. Additionally, elevated pro-inflammatory cytokine levels, reduced interferon-γ (IFN-γ) levels, and reduced CD4^+^ and CD8^+^ T cells suggest that the immune system is dysregulated in COVID-19 with a positive correlation between severity of symptoms and the extent of dysregulation [94,95]. Many months after COVID-19 was considered a pandemic, promising therapeutic and prophylactic pharmacological agents received emergency authorization, but a cure per se has not yet been established. In this regard, PA as a nonpharmacological modality that can help to enhance the immune and musculoskeletal systems if performed safely at an optimal intensity and duration (Figure 1, Figure 2 and Figure 3).

There is a strong association between the type of PA and benefits to the immune system. PA, such as Pilates training performed for 180 min per week, during two weeks of acute PA, improves the innate immune response in adult women, as detected by increased NK cell lytic activity and decreased monocyte chemotactic protein-1 (MCP-1) [96,97]. Bicycle ergometry performed for six minutes at 55% of VO_2_ max or for 30 min at 11.11 km/h increases the number of leukocytes (by ~36%), granulocytes (by ~29%), lymphocytes (by ~46%), and monocytes (by ~68%) in circulating blood [78,80]. An acute bout of PA increases circulating concentrations of CD4+ lymphocytes (by 30–40%) and CD8+ lymphocytes (by 90–105%) in peripheral blood [97]. Moderate intensity PA reduces toll-like receptors (TLR), TLR2 (by ~35%), TLR4 (by ~25%), and IL-6 (by ~20%) [84]. After moderate treadmill aerobic training or resistance training that was performed three times per week for three months, blood concentrations of pro-inflammatory markers TNF-α, IL-2, IL-4, and CRP were reduced in women [98]. Moderate PA and improved CRF reduce CRP levels and might, therefore, be beneficial for patients with COVID-19 [98,99]. Moderate PA can also help reduce tissue oxidative stress, which in turn reduces inflammation [83]. Thus, due to immune system modulating effects, moderate PA during the COVID-19 pandemic might be beneficial for both healthy women as well as women with asymptomatic COVID-19 infection [75].

IL-6 is a cytokine that has a dual role, in that, it exerts pro-inflammatory effects when released by inflammatory cells and anti-inflammatory effects when released by skeletal muscle [100]. During PA, contracting muscles release IL-6 into the circulation, which acts as an endocrine signal and exerts positive effects on multiple target tissues [76,80]. IL-6 release from skeletal muscles is linked to glycogen depletion in muscle, which is in contrast to what is observed in COVID-19 and related diseases, where IL-6 elevation is a result of injury and inflammation in infected cells/tissues [101,102]. PA-induced elevation in blood IL-6 levels is transient and returns to resting levels usually within a few hours after PA, whereas IL-6 elevation may persist for many days with tissue injury and inflammation [99].

## 4. The Specific Role of Mucosal Immunity and Immunoglobulin A (IgA) in Protection against Respiratory Infections and Symptoms

It is well known that the mucosal immune system provides resistance to the upper respiratory tract infection (URTI), primarily through airway secretory immunoglobulin A (abbreviated as SIgA or S-IgA; sometimes referred to as salivary IgA and abbreviated s-IgA when measured in saliva) [103,104,105]. SIgA represents one of the body’s first lines of defense against URTI through its capacity to inhibit pathogen colonization, bind antigens for transport across epithelial barriers, and neutralize viruses [105,106]. It is now well established that high PA loads (e.g., marathon running) tend to decrease SIgA levels and, thus, render individuals more susceptible to upper respiratory illness (URI) and upper respiratory symptoms (URS), while moderate PA loads tend to increase SIgA levels, thus providing a first line of defense against URS [107,108] (Figure 2).

The role of PA levels on SIgA is relevant to COVID-19 since emerging data suggest a link between SIgA and SARS-CoV-2. Ejemel and colleagues found that an IgA form of an antibody raised against the SARS-CoV-2 spike protein showed superior target binding and virus neutralization when compared to its IgG counterpart [109]. Since mucosal SIgA exists mostly in a dimeric form, Wang and colleagues compared the neutralizing effects of monomeric and dimeric forms of anti-SARS-CoV-2 IgA and found that the dimeric form is ~15-fold more effective in neutralizing SARS-CoV-2 than the monomeric form and is also several-fold more effective than anti-SARS-CoV-2 IgG [110]. Sterlin and colleagues studied samples from patients with COVID-19 and found the following: anti-SARS-CoV-2 IgA antibody levels rise and fall earlier than IgG antibody levels; IgA preparations were more effective than IgG preparations in neutralizing SARS-CoV-2 pseudovirus; anti-SARS-CoV-2 IgA levels positively correlated with virus neutralization; and anti-SARS-CoV-2 IgAs in bronchoalveolar lavage preparations were more effective in pseudovirus neutralization than compared to their IgG counterparts—all suggesting that IgA-based mucosal immunity likely plays a role in countering SARS-CoV-2 [111]. The relevance of IgA-mediated immunity relative to vaccine-induced protection against COVID-19 is, thus, obvious [112,113]. However, PA-induced SIgA changes, which correlate with protection against URI, and URS must also be emphasized since all individuals might not respond in the same manner with respect to vaccines (e.g., immunocompromisation and immunosenescence) [114] and because vaccine eligibility and supplies are affected by individual, social, political, and economic factors [115,116]. Several studies investigating the elderly have demonstrated that SIgA levels and secretion rates increase with many weeks to months of moderate intensity PA, which includes both strength and endurance training, thus suggesting that the effects of immunosenescence could somewhat be countered by consistent PA in this population [117,118]. Although the positive effects of PA on SIgA and the benefits of SIgA in defense against URI and URS are known, at this time, it is unknown as to whether or not PA specifically improves mucosal immunity against SARS-CoV-2 in either women or men.

## 5. Women, PA, and Post-Acute Sequelae of SARS-CoV-2 Infection (PASC)

COVID-19 infection rates appear to be similar between females and males in young, asymptomatic populations [119], as well as in older symptomatic cohorts around the world [86]. Mortality rates from acute COVID-19 infections are higher in males [74,86,88], while chronic illness (i.e., PASC also known as “long COVID” or “long haul COVID”) rates are higher among females [31,120,121,122,123]. PASC is associated with symptoms, such as physical and cognitive fatigue, breathing difficulty, gastrointestinal disturbances, and changes in mental health, which can persist for many months after COVID-19 infection [30,31]. The debilitating consequences of PASC underscore the need for both men and women to engage in regular, moderate PA in order to maximize mental, physical, and immunological health. However, the health and societal burden from chronic functional impairments associated with PASC appear to fall disproportionately on women, which may have potentially devastating downstream effects on individuals, families, and societies far beyond the acute phase of SARS-CoV-2 infection [120,121,122,123].

The potential for long-term illness following acute SARS-CoV-2 infection is supported by longitudinal studies on survivors of SARS-CoV-1 infection, which was responsible for the original SARS outbreak of 2003 [31,124]. In one cohort of 233 survivors hospitalized in Hong Kong, 40% reported the persistence of at least one psychiatric illness, while 40.3% reported chronic fatigue based upon a survey conducted four years after acute SARS illness [123]. Furthermore, healthcare workers were at increased risk for psychiatric symptoms (odds ratio 3.24), while females were overrepresented as study participants (70.4%) [123]. Clinical interviews (performed on 181 of 233 survivors) revealed that 46.2% of participants with persistent psychiatric symptoms remained unable to work at the 4-year follow-up. Only 3.3% had a prior history of psychological disturbances before SARS, and they still had ongoing psychological symptoms four years after acute illness, which included post-traumatic stress disorder (in 54.5%), depression (in 39%), somatoform pain disorder (in 36.4%), panic disorder (in 32.5%), and obsessive-compulsive disorder (in 15.6%) [123].

Both SARS-CoV-1 and SARS-CoV-2 are beta coronaviruses, which are positive-sense, single-stranded RNA viruses, and enter host cells through ACE2 [102,124,125,126,127,128]. The respective spike (S) proteins of SARS-CoV-1 and SARS-CoV-2, which decorate the surface of viral particles and give these viruses with their characteristic solar corona-like appearance, share ~75% sequence homology [124]. The ~25% difference in the S protein between SARS-CoV-1 and SARS-CoV-2 could be responsible for differences between SARS and COVID-19 (e.g., symptomatic virus shedding from the lower airways in SARS-CoV-1 versus asymptomatic virus shedding from the upper airways in SARS-CoV-2), and the ~75% homology could explain why both diseases are highly contagious and are associated with high case fatality in people ≥50 years) [129]. Based on post-SARS-CoV-1 infection data, the potential for similar lingering symptoms following COVID-19—particularly in women—appears to be high and exacerbated by the sustained numbers of new SARS-CoV-2 infections around the globe [31].

The potential for debilitating fatigue, psychiatric illness, and neurological complaints following COVID-19 infection is physiologically supported by laboratory studies. Translocation of the S protein from SARS-CoV-2 from the systemic circulation into the brain occurs via adsorptive transcytosis across the blood–brain barrier (BBB) in murine models [130]. Additionally, in vitro studies suggest that SARS-CoV-2 can replicate within neuronal cells [131]. Collectively, it appears that SARS-CoV-2 infection, through direct and indirect effects on the brain and other neural tissues, may cause a variety of neurological and psychological manifestations that are common in PASC (e.g., fatigue and “brain fog”) [31,123]. The emergence of PASC highlights the effects of sex as a biological variable in COVID-19 [31,86,132]. Females mount a stronger innate, cellular, and humoral immune response to viral infections but are at higher risk for chronic autoimmune and immunogenic disorders [87,132]. Thus, although men are at a higher risk than women for severe illness and death from acute COVID-19, women are at a greater risk for chronic COVID-19 illness due to PASC [31,86].

It is important to note that post-viral fatigue is not specific to SARS and COVID-19, as chronic fatigue syndromes are well described following infections caused by influenza viruses (H1N1), Epstein–Barr virus, Ebola virus, and West Nile virus [133]. The common theme of post-viral fatigue, regardless of the pathogen, is that it is more frequent, severe, and prolonged in women than in men [120,121,122,123]. For this reason, physicians [134] and scientists [135] are linking the pathogenesis and clinical signs and symptoms of PASC with a similar disabling condition known as myalgic encephalomyelitis/chronic fatigue syndrome (ME/CFS) [136]. ME/CFS also disproportionately affects women (especially White/Caucasian women) and was first described as “yuppy flu” in the 1980s due to its poorly understood psychological and physical (e.g., fatigue) manifestations [135]. The pathophysiology of ME/CFS is characterized by autoimmunity and low-grade inflammation resulting from elevated oxidative and nitrosative stress (O&NS), mitochondrial dysfunction, and activation of pro-inflammatory pathways [135,137].

The early signs and symptoms of PASC mimic ME/CFS, and emerging studies confirm that females are overrepresented in cohorts with lingering post-COVID symptoms [120,121,122,123]. What is most concerning, however, is the growing scientific recognition that persistent fatigue, neurological manifestations, and PA intolerance occur independent of symptom severity and age [121,138]. Recent reports document PASC in ~51% of individuals in a cohort of 43 COVID-19-positive college-students (96% female) with mild symptoms [121], as well as in five children between the ages of 9 and 15 (80% female) [139]. Since PASC can affect females from a diverse range of populations (e.g., healthcare and services sector workers, primary caregivers to dependent children and others, teachers, and school-age children), it is of great population health and socioeconomic concern.

A worrisome hallmark of ME/CFS is PA intolerance, wherein even slightly excessive PA appears to exacerbate symptomatology or precipitate a relapse into chronic fatigue [135,140]. Curiously, overtraining syndrome mimics both ME/CFS and PASC, suggesting that overlapping neuro-inflammatory, autoimmune, and/or autonomic pathophysiological processes might be at play [141] (Figure 3). Thus, the true paradox of PA and COVID-19 is that although mild to moderate regular PA may prevent or attenuate morbidity and mortality from COVID-19, once infected with SARS-CoV-2 and PASC develops, the positive health benefits of PA may be negated [121,122,138,139,140]. Future longitudinal investigations are required to further dissect the effects of sex as a biological variable in the effect of PA as a preventive and/or remedial measure against acute and chronic COVID-19 illness. At present, it appears that in untrained individuals and in individuals with compromised PA tolerance, intense and fatiguing PA in the context of acute or chronic COVID-19 illness may be detrimental, but mild to moderate PA that is adjusted based on RPE might be beneficial for optimizing mental, physical, metabolic, and immune health (Figure 2 and Figure 3). As with ME/CFS, the most practical strategy to work through PASC might be to balance PA with intentional rest, avoid fatigue, pace daily routines, and resist the urge to “push oneself physically” on good days [142,143]. Additionally, due to the gravity of acute COVID-19 and PASC, it is highly recommended that women receive prophylactic pharmacological therapy against acute COVID-19 as early as possible (i.e., vaccination when eligible) and to continue to follow nonpharmacological physical risk mitigation measures (distancing, masking, hygiene, and self-isolation when sick) as part of maintaining an active lifestyle that prioritizes optimal PA (Figure 3).

## 6. Recommendations for Staying Active during a the COVID-19 Pandemic

The WHO campaign “Be active and Stay Healthy at home”, in accordance with the PA Guidelines for Americans, recommends performing adequate PA to improve the following: physical fitness (cardiorespiratory and muscular fitness), cardiometabolic fitness (blood pressure, lipid profile, and glycemic control), bone health, cognitive outcomes and mental health, balance, and flexibility [19,20,21,29]. For adults between 18 and 64 years, 150–300 min of moderate intensity PA or at least 75–150 min of vigorous PA throughout the week is recommended. Pregnant healthy women should undertake at least 150 min per week of moderate intensity cardiorespiratory (i.e., aerobic) PA in order to increase or maintain CRF, optimize BMI, and reduce the risk and severity of postpartum depression [21]. If pregnant women were accustomed to vigorous aerobic PA before pregnancy, they may continue that level of PA during pregnancy [19]. The recommendation for girls between 6 and 17 years is to perform an average of 60 min of moderate to vigorous PA per day. For older adults, it is suggested that they engage in moderate intensity PA for >3 days each week and to undertake up to 300 min of PA per week in order to enhance functional capacity and prevent falls [19].

The American College of Sports Medicine (ASCM) has published specific guidelines on how to remain physically active during the COVID-19 pandemic [144,145]. There is no recommendation at this time to limit PA during acute uncomplicated COVID-19. However, in light of what is known (and has been discussed in preceding sections of this paper) about the effects of PA on the immune system, it seems logical that unaccustomed and intense PA may not be advisable in order to avoid overwhelming the immune and other physiological systems. Furthermore, when COVID-19 is suspected or confirmed, it is necessary to monitor symptoms (mainly difficulty breathing and reduced oxygen saturation measured with a finger pulse oximeter) and assiduously follow physical risk mitigation measures (distancing, masking, hygiene, and self-isolation) in order to avoid complications and reduce community spread of SARS-CoV-2 through aerosolized viral particles [3,146].

Regular, moderate intensity PA provides numerous mental and physical health benefits to women in the context of COVID-19 or otherwise (Figure 1). However, the complex roles played by women in society can render it challenging for them to be motivated to consistently make PA a priority [53,147]. Women are likely to adhere to regular PA routines when there is social support, while men may rely more on competition to keep them motivated [148]. While there is no doubt that performing optimal levels of PA during the COVID-19 pandemic might be challenging, receiving prophylactic pharmacological therapy against serious complications as early as possible (i.e., vaccination when eligible) and continuing to follow nonpharmacological physical risk mitigation measures (distancing, masking, hygiene, and self-isolation when sick) make the goal of consistently engaging in moderate-intensity PA quite achievable (Figure 3).

## 7. COVID-19 Vaccination and Its Relevance to Women’s Health and Maintaining an Active Lifestyle

As of 26 July 2021, a conservative estimate of total COVID-19 cases was ~194 million people, of which four million people had died, thus placing the worldwide case fatality rate at ~2% (about one death for every 50 confirmed cases) [149]. When COVID-19 was declared a global pandemic in 2020, clinicians and scientists around the world desperately looked to find therapeutics that could be repurposed to reduce the rapidly rising number of COVID-19 deaths [102,150,151,152,153]. In less than a year, through the concerted and concurrent efforts of health agencies, scientists, clinicians, industry partners, and research volunteers worldwide, many vaccine candidates were developed and tested. Vaccines that passed rigorous preclinical testing (testing in animals) and phased clinical trials (testing in humans) and that were deemed safe (i.e., extremely rare serious side effects and adverse events) and effective (i.e., reduced the probability of infection and serious illness) by multiple regulatory agencies were granted emergency use authorization [154,155,156,157]. Emerging data indicate that the widely administered BNT162b2 vaccine (mRNA technology; manufactured by: Pfizer, New York, NY, USA, and BioNTech, Maintz, Germany) and ChAdOx1 nCoV-19 vaccine (adenoviral vector technology; manufactured by: Oxford University, Oxford, UK, and AstraZeneca, Cambridge, UK) are effective at reducing infection [158] and hospitalization [159], even against new and highly contagious SARS-CoV-2 variants (e.g., the Delta strain). Thus, it can be concluded that the best method to prevent hospitalization and death from COVID-19 would be to receive one of the vaccines that have been recognized by a reputable health agency, such as the WHO [160]. The unprecedented ability to receive protection against COVID-19 hospitalization and death has unfortunately been undermined by rampant misinformation regarding COVID-19 [161,162] coupled with vaccine inequity and ineligibility worldwide [116,163]. Receiving a vaccine as soon as possible when eligible will help women engage more safely in PA due to the reduced risk of acute COVID-19 infection and complications if exposed to SARS-CoV-2 (Figure 3).

With regards to COVID-19 vaccination and women’s health, data from the United States collected during the first month of the vaccine rollout when only mRNA vaccines were available showed that more women (61.2%) received vaccination compared to men [164]. However, a greater proportion of women also reported side effects or adverse events (78.7%) after receiving a vaccine [164]. Even though women report side effects or adverse events more frequently after receiving an mRNA COVID-19 vaccine, the protection rendered against acute COVID-19, with or without PASC, far exceeds the transient side effects. Further investigation is warranted on sex-specific, post-vaccination symptomatology, immunological responses, and the risk of breakthrough infection and transmission [165]. However, at this time there are no scientific data to support concerns regarding derangements in menstruation, fertility, childbearing capabilities, or an increased risk with respect to pregnant women or the developing fetus following vaccination [164,165]. It could be argued that the safety and adverse event profiles of the authorized vaccines are even better than some of the commonly used over-the-counter medications, such as nonsteroidal anti-inflammatory drugs (NSAIDs; e.g., drugs that end with the suffix -profen, -proxen, -oxicam, and -fenac), that are taken for musculoskeletal pain [166]. Gaining vaccine confidence in women might have widespread global health benefits due the pivotal role they play in the health and wellness of families through nurturing and caregiving for dependent children and others [167]. Nonetheless, women must be allowed to make independent and informed decisions regarding receiving COVID-19 vaccination in consultation with their healthcare providers—this must be based on best medical practices and not based on misinformation and societal pressure [168]. Finally, women should be able to rest and slowly ramp up PA based on how their body responds to COVID-19 vaccination [169]. Some nations, such as New Zealand, have implemented leave policies for individuals who might develop a rare adverse reaction following COVID-19 vaccination [170] (Supplementary Materials—personal correspondence from Mr. Moses Benjamin, Allied Health Director, Auckland District Health Board, New Zealand).

Despite the protection against COVID-19 hospitalization and death offered by vaccines, the WHO is requesting nations where vaccination rates are high to continue to follow nonpharmacological physical risk mitigation measures, such as social distancing, wearing proper facemasks, following good sanitation and hygiene practices, and getting tested and self-isolating when sick [171]. The need for continued physical risk mitigation measures even after vaccination is supported by mathematical models, which suggest that, even with perfect vaccine acceptance scenarios, it would likely take many months to a year for community spread to consistently remain at low levels that are not of concern [24,25]. The risk of rare breakthrough infections (i.e., vaccinated individuals that are infected with SARS-CoV-2) [172,173], the possibility of new viral variants emerging in unvaccinated and vaccinated individuals due to the inherent biology of coronaviruses [174], and the fact that only a few countries currently have enough doses to vaccinate their populations [175] collectively validate the WHO’s abundance of caution and related recommendations [171]. Since the health of individuals in any part of the world has an impact on global health, all nations must show solidarity with the rest of the world and follow the WHO’s recommendations in order to aggressively vaccinate their populations, share unused vaccine doses with other countries, and continue to follow physical risk mitigation measures in order to complement worldwide vaccination efforts. The slogan of COVAX, the WHO-led alliance for global equitable access to COVID-19 vaccines, sums it best: “with a fast-moving pandemic, no one is safe, unless everyone is safe” [176].

## 8. Conclusions

PA during the COVID-19 pandemic is a double-edged sword for women since mild to moderate PA (based on RPE) may be beneficial, but unaccustomed and intense PA could increase illness risk. Moderate PA may enhance immune and other physiological functions, but intense PA is best avoided by untrained individuals because it may trigger maladaptive physiological responses, rendering people more susceptible to acute and chronic COVID-19 complications. Although SARS-CoV-2 is likely to infect women and men at similar rates, sex-specific behavioral and physiological responses may alter the clinical trajectory of COVID-19, e.g., higher risk of acute illness complications in men but higher incidence and severity of PASC in women may occur. From a mental health perspective, it is clear that women, as caregivers, are disproportionally overburdened by mental health crises. Depression, emotional stress, anxiety, eating disorders, and psychoactive substance cravings are reduced by regular PA and, therefore, should be encouraged in order to improve both mental and physical health. Pandemic precautions must, however, be followed diligently to keep oneself safe and to minimize community spread of SARS-CoV-2. Moving forward, investigations on the influence of sex hormones on PA-induced immunomodulation may identify physiological responses that may be protective against COVID-19 (and offer therapeutic targets). Due to the novelty of SARS-CoV-2 in humans, comprehensive clinical studies, follow-up cohort assessments, and analyses of data in a sex-disaggregated manner are needed for elucidating the effects of preventive interventions (e.g., PA) when pandemic precautions are in effect. Finally, as part of prioritizing an active lifestyle, it is essential that women receive prophylactic pharmacological therapy against serious complications as early as possible (i.e., vaccination when eligible) and continue to follow nonpharmacological physical risk mitigation measures (social distancing, masking, hygiene, and self-isolation when sick). Such healthy behaviors will contribute to personal, family, community, and global health and wellness, and will ultimately accelerate exiting the COVID-19 pandemic.

## Data Availability

Data sharing not applicable. No new data were created or analyzed in this study. Data sharing is not applicable to this article.

## Supplementary Materials

The following are available online at https://www.mdpi.com/article/10.3390/ijerph181910271/s1, File S1: Personal correspondence from Moses Benjamin, Allied Health Director, Auckland District Health Board, New Zealand, regarding New Zealand’s leave policy for employees who experience side effects and/or adverse events following COVID-19 vaccination.
