# Supplementary material for: Women’s Lives Matter—The Critical Need for Women to Prioritize Optimal Physical Activity to Reduce COVID-19 Illness Risk and Severity
Source: Int J Environ Res Public Health. 2021 Sep 29;18(19):10271. doi: 10.3390/ijerph181910271 (PMC8507774; doi:10.3390/ijerph181910271)
Supplement: Supplementary file 1 [file ijerph-18-10271-s001.zip › ijerph-1348908-supplementary.pdf]

Regarding your enquiry

Moses Benjamin (ADHB) <MosesB@adhb.govt.nz>
Thu 5/13/2021 12:48 AM
To: Joseph Roche <joseph.roche@wayne.edu>

[EXTERNAL]

Der Roche

Thank you for your query relating to sick /rest management post vaccination.

We have a National Policy in place currently as below:

↓ What happens I have a reaction?

Some people may experience a mild reaction to the COVID-19 vaccination. Where they are injected they may experience soreness, redness, warmth and slight swelling. These effects usually last less than two days and should not stop you from attending work.

However, in the unlikely event that you do require time off work due to the effects of the vaccine, please advise your manager that this should be on paid special leave (PDSP in Workforce Central).

Workforce Central is the platform for recording leave/pay etc.
I hope this helps.

Warm Regards

Moses Benjamin
Allied Health Director
Interim General Manager, Pathology & Laboratory Medicine
Clinical Support Directorate
(09 307 4949 X 22803 |(021 227 3891 | Ê 09 307 8987 |* mosesb@adhb.govt.nz
Te Toka Tumai | Auckland District Health Board | Level 5 |Building 32 | Pvt. Bag 92024 | Auckland City Hospital

Te tino o mātou – *Us at our best* AUCKLAND

Welcome *Haere Mai* | Respect *Manaaki* | Together *Tūhono* | Aim High *Angamua*

The information contained in this email and any attachments is confidential and intended for the named recipients only. If you are not the intended recipient, please delete this email and notify the sender immediately. Auckland DHB accepts no responsibility for changes made to this email or to any attachments after it has been sent.

Dear Moses,
Greetings!
As indicated in our brief WhatsApp conversation, a few colleagues and I are writing a paper on "just right physical activity" in the context of preventing complications from COVID-19 illness and vaccination. Since you are in a leadership position for your country's department of health, I would be very grateful if you may please be able to share information on trends you are observing with reference to individuals requiring a sick/rest day following COVID-19 vaccination. In my paper, I plan on making the case that, employers should make contingencies to accommodate the need for a sick/rest day for their employees following vaccination, as part of reducing the risk of post vaccination complications.
In my paper, I will cite the information you share as personal correspondence and mention your name and designation. I would alse be happy to mention you in the list of acknowledgments in the paper.
Thank you so very much.

Sincerely,
Roche

Dr. Joseph A. Roche, BPT, Dip. Rehab. PT, PhD
Pronouns: he, him, his
Associate Professor
Physical Therapy Program
Wayne State University
259 Mack Avenue, Rooms 2339 (office) and 4440 (lab)
Detroit, MI 48201
Phone: 443-413-9494
Email: joseph.roche@wayne.edu

Wayne State University's Mission is: To create and advance knowledge, prepare a diverse student body to thrive, and positively impact local and global communities
